# One-lung ventilation patients: Clinical context of administration of different doses of dexmedetomidine

**DOI:** 10.5937/jomb0-33870

**Published:** 2022-04-08

**Authors:** Hui Jiang, Yu Kang, Chunlin Ge, Zhenying Zhang, Yan Xie

**Affiliations:** 1 Xuhui District Central Hospital, Department of Anesthesiology, Shanghai, China; 2 Shanghai Jiao Tong University School of Medicine, Tongren Hospital, Department of Anesthesiology, Shanghai, China

**Keywords:** dexmedetomidine, one-lung ventilation, inflammatory response, oxidative stress, cerebral tissue oxygen saturation, intrapulmonary shunt, deksmedetomidin, ventilacija jednog plućnog krila, inflamatorni odgovor, oksidativni stres, saturacija cerebralnog tkiva kiseonikom, intrapulmonalni šant

## Abstract

**Background:**

Open and endoscopic thoracic surgeries improve surgical exposure by One-lung ventilation (OLV). The aim of this study was to investigate the effects of different doses of dexmedetomidine on inflammatory response, oxidative stress, cerebral tissue oxygen saturation (SctO2) and intrapulmonary shunt in patients undergoing one-lung ventilation (OLV).

**Methods:**

Seventy-five patients undergoing open pulmonary lobectomy in our hospital from January 2016 to December 2017 were enrolled and randomly divided into high-dose dexmedetomidine group (group D1, 1 mg/kg, n=25), low-dose dexmedetomidine group (group D2, 0.5 mg/kg, n=25) and control group (group C, n=25). Then, arterial blood and internal jugular venous blood were taken before anesthesia induction (T0) and at 15 min after twolung ventilation (T1) and 5 min (T2) and 30 min (T3) after OLV for later use. Next, the changes in hemodynamic parameters [mean arterial pressure (MAP), heart rate (HR) and pulse oxygen saturation (SpO2)] of patients were observed in each group. Enzyme-linked immunosorbent assay (ELISA) was carried out to detect serum inflammatory factors such as interleukin-6 (IL-6) and tumor necrosis factor-alpha (TNF-α) and oxidative stress indicators [superoxide dismutase (SOD) and malondialdehyde (MDA)]. The changes in SctO2, arterial partial pressure of oxygen (PaO2) and intrapulmonary shunt Qs/Qt (a measurement of pulmonary shunt: right-to-left shunt fraction) were observed. Additionally, the changes in lung function indicators like lung dynamic compliance (Cdyn) and airway peak pressure (P_peak_) were determined.

**Results:**

There were no statistically significant differences in the MAP, HR and SpO2 among three groups at each observation time point (P>0.05). At T2 and T3, the levels of serum IL-6, TNF-α and IL-8 were obviously decreased in group D1 and D2 compared with those in group C (P<0.05), and the decreases in group D1 were overtly larger than those in group D2, and the decreases at T3 were markedly greater than those at T2 (P<0.05). In comparison with group C, group D1 and D2 had notably reduced levels of serum reactive oxygen species (ROS) and MDA (P<0.05) and remarkably increased SOD content (P<0.05) at T2 and T3, and the effects were markedly better in group D1 than those in group D2. Besides, they were significantly superior at T3 to those at T2 (P<0.05). The SctO2 in group D1 and D2 was evidently lowered at T2 and T3 compared with that at T0, and the decrease in group D1 was distinctly smaller than that in group D2 (P<0.05). The Qs/Qt was significantly lower in group D1 and D2 than that in group C at T2 and T3 (P<0.05), while the PaO2 content was notably raised (P<0.05), and the decrease and increase were significantly larger in group D1 than those in group D2, and they were obviously greater at T3 to those at T2 (P<0.05). At T0 and T1, no significant differences were detected in the Cdyn, Pplat and Ppeak among three groups. At T2 and T3, the Cdyn was significantly elevated, while the Pplat and Ppeak overtly declined (P<0.05), and group D1 had greater changes in comparison with group D2, and the changes were obviously more evident at T3 to those at T2 (P<0.05).

**Conclusions:**

Dexmedetomidine effectively ameliorates inflammatory response and oxidative stress, lowers oxygenation, Qs/Qt and the decrease in SctO2 and improves lung function during OLV, with good efficacy.

## Abstract (sr)

**Uvod:**

Otvorene i endoskopske torakalne operacije poboljšavaju hirurško izlaganje pomoću ventilacije jednog plućnog krila (OLV). Cilj ove studije je bio da se ispitaju efekti različitih doza deksmedetomidina na inflamatorni odgovor, oksidativni stres, zasićenost cerebralnog tkiva kiseonikom (SctO2) i intrapulmonalni šant kod pacijenata koji su podvrgnuti ventilaciji jednog pluća (OLV).

**Metode:**

Sedamdeset pet pacijenata koji su bili podvrgnuti otvorenoj plućnoj lobektomiji u našoj bolnici od januara 2016. do decembra 2017. godine upisani su i nasumično podeljeni u grupu sa visokim dozama deksmedetomidina (grupa D1, 1 mg/kg, n=25), niskom dozom deksmedetomidina (grupa D2), 0,5 mg/kg, n=25) i kontrolna grupa (grupa C, n=25). Zatim su uzete arterijska krv i unutrašnja jugularna venska krv pre indukcije u anesteziju (T0) i 15 min nakon ventilacije sa dva plućna krila (T1) i 5 min (T2) i 30 min (T3) nakon OLV za kasniju upotrebu. Zatim su uočene promene hemodinamskih parametara [srednji arterijski pritisak (MAP), broj otkucaja srca (HR) i pulsna zasićenost kiseonikom (SpO2)] pacijenata u svakoj grupi. Enzimski imunosorbentni test (ELISA) je sproveden da bi se otkrili serumski inflamatorni faktori kao što su interleukin-6 (IL-6) i faktor nekroze tumora-alfa (TNF-a) i indikatori oksidativnog stresa [superoksid dismutaza (SOD) i malondialdehid (MDA)]. Uočene su promene u SctO2), arterijskom parcijalnom pritisku kiseonika (PaO2) i intrapulmonalnom šantu Ks/Kt (merenje plućnog šanta: frakcija šanta zdesna nalevo). Pored toga, određene su promene u indikatorima plućne funkcije kao što su dinamika plućne usklađenosti (Cdin) i vršni pritisak u disajnim putevima (Ppeak).

**Rezultati:**

Nije bilo statistički značajnih razlika u MAP, HR i SpO2 između tri grupe u svakoj vremenskoj tački posmatranja (P>0,05). Na T2 i T3, nivoi serumskih IL-6, TNF-a i IL-8 su očigledno bili smanjeni u grupi D1 i D2 u poređenju sa onima u grupi C (P<0,05), a smanjenje u grupi D1 je bilo očigledno veće od oni u grupi D2, a smanjenja na T3 bila su značajno veća od onih u T2 (P<0,05). U poređenju sa grupom C, grupe D1 i D2 su imale značajno smanjene nivoe serumskih reaktivnih vrsta kiseonika (ROS) i MDA (P<0,05) i značajno povećan sadržaj SOD (P<0,05) na T2 i T3, a efekti su bili znatno bolji u grupi D1 od onih u grupi D2. Osim toga, oni su bili značajno bolji na T3 u odnosu na one na T2 (P<0,05). SctO2 u grupi D1 i D2 je evidentno sma - njen na T2 i T3 u poređenju sa onim u T0, a smanjenje u grupi D1 je bilo znatno manje nego u grupi D2 (P<0,05). Ks/Kt je bio značajno niži u grupi D1 i D2 nego u grupi C na T2 i T3 (P<0,05), dok je sadržaj PaO2 bio značajno povišen (P<0,05), a smanjenje i povećanje su značajno veće u grupi. D1 od onih u grupi D2, i očigledno su bili veći na T3 u odnosu na one u T2 (P<0,05). Na T0 i T1, nisu otkrivene značajne razlike u Cdin, Pplat i Ppeak između tri grupe. Na T2 i T3, Cdin je bio značajno povišen, dok su Pplat i Ppeak izrazito opali (P<0,05), a grupa D1 je imala veće promene u poređenju sa grupom D2, a promene su očigledno bile očiglednije na T3 u odnosu na one na T2 ( P<0,05).

**Zaključak:**

Deksmedetomidin efikasno ubla'ava inflamatorni odgovor i oksidativni stres, smanjuje oksigenaciju, Ks/Kt i smanjenje SctO2 i poboljšava funkciju pluća tokom OLV, sa dobrom efikasnošću.

## Introduction

One-lung ventilation (OLV) is commonly used for open and endoscopic thoracic surgeries to improve surgical exposure. Hypoxic pulmonary vasoconstriction (HPV) shunts blood from the non-ventilated lung to the non-surgical lung, thus maintaining adequate oxygenation [1] [2]. Thoracic epidural anesthesia (TEA) is the most commonly used analgesia technique in patients receiving thoracic surgeries for lungs. It has been proved that strong inhalation anesthesia inhibits HPV in a dose-dependent manner, thereby altering the oxygenation during OLV and resulting in increased shunt and impaired oxygenation [3] [4]. Studies have revealed that the development and progression of inflammatory response and oxidative stress are promoted in the peri-operative period. Dexmedetomidine is a highly selective and very potent α2-adrenergic agonist with antioxidant properties, metabolized in the liver and approved to be used in intensive care units as a sedation and anesthesia assistor, with sedative and analgesic effects [5].

A previous study demonstrated that dexmedetomidine lowers the high levels of malondialdehyde (MDA) and hypoxanthine formed after the application of tourniquets in upper-limb surgeries, with an obvious effect [6]. Dexmedetomidine has analgesic, anxiolytic and sedative effects, on which many clinical and experimental studies have been conducted in recent years [7] [8]. Moreover, research of the role of dexmedetomidine in ischemic and toxic inflammation models has revealed that dexmedetomidine has an anti-inflammatory effect, which evidently inhibits the production of inflammatory factors including tumor necrosis factor-alpha (TNF-α), interleukin-1β (IL-1β), IL-6 and macrophage inflammatory protein 2, and avoids damage to organs. Furthermore, it has been reported that dexmedetomidine prominently relieves lung inflammation in a rat model of ventilatorinduced lung injury. Shen et al. [9] measured the levels of TNF-α and IL-6 in the case of lung injury induced in experimental models and proved that the production of these cytokines is reduced to the utmost in lung tissues after administration with dexmedetomidine. A study pointed out that mouse liver ischemia-reperfusion (I/R) injury results in oxidative stress, which is manifested as enhanced MDA, an oxidant and reduced superoxide dismutase (SOD), an antioxidant [10]. Lung tissues are vulnerable to the harmful effects of hypovolemia, and excessive inflammation and oxidative stress response are detected in a mouse model, including, including SOD and MDA [11]. SOD is ubiquitous, and MDA can resist the effects of SOD, with cytotoxicity. After I/R, the treatment with the antioxidant dexmedetomidine is capable of ameliorating organ oxidative stress and achieving better outcomes. Antioxidant therapy with transmembrane free radical scavengers can improve the prognosis of I/R rats [12]. The above findings suggest that dexmedetomidine effectively attenuates inflammatory response and oxidative stress during surgery. The changes in cerebral tissue oxygen saturation (S_ct_O_2_) are measured horizontally, continuously and noninvasively in the peri-operative period through the frontal microvascular system. Additionally, the specific changes in S_ct_O_2_ are monitored to provide real-time oxygenation in local tissues during full-circulation arrest, venous cannula obstruction or sudden global hypoxemia in cardiac surgeries. Reduced S_ct_O_2_ is considered as an indication of potential hypoxiainduced injury that needs further interventions [13]. Evaluating cardiac output and systemic oxygenation sufficiency may have potential value in changing the ventilation mode in the peri-operative period in patients who received the bidirectional Glenn procedure [14].

This study aims to explore the effects of different doses of dexmedetomidine on inflammatory response, oxidative stress, S_ct_O_2_ and intrapulmonary shunt in patients receiving OLV. Patients undergoing open pulmonary lobectomy were enrolled in this study, and then hemodynamic parameters, inflammatory factors, oxidative stress indicators, and changes in the S_ct_O_2_, arterial oxygen partial pressure (PaO_2_) and Qs/Qt as well as lung function were observed at different time points, hoping to prove that dexmedetomidine can effectively relieve inflammatory response and oxidative stress, lower oxygenation and Qs/Qt and improve S_ct_O_2_ and lung function during OLV, with good effects. This study provides theoretical and experimental bases for the popularization and application of dexmedetomidine.

## Materials and Methods

### Clinical data

Seventy-five patients who underwent open pulmonary lobectomy in our hospital were enrolled as study subjects. Then, the patients enrolled signed the informed content and were randomly divided into high-dose dexmedetomidine group (group D1, 1 μg/kg, n=25), low-dose dexmedetomidine group (group D2, 0.5 μg/kg, n=25) and control group (group C, n=25). Inclusion criteria: Patients at America Society of Anesthesiologist (ASA) grade I and II, receiving no treatment previously, and not allergic to the drugs used in this study. Exclusion criteria: Patients allergic to the drugs, or with severe cardiovascular or cerebrovascular diseases, or secondary infection complicated with severe abnormal liver or kidney function, or those unable to communicate normally due to severe mental disorders. All clinical specimens in this study were collected with the consent of the patients and their families as per the *Declaration of Helsinki*. This clinical study protocol was carried out with approval from the Ethics Committee of our hospital. The specific clinical data of patients collected at admission included age, gender, weight, body condition and pathological grade (Table 1).

**Table 1:** Clinical data of patients

Parameter	Group D1	Group D2	Group C
Sample size	25	25	25
Number of male patients	12	13	12
Average age (years old)	45±11	46±12	47±11
Mean weight (kg)	49±10.5	50±10	48±10.7
BMI (kg/m^2^)	21.5±3.2	22.1±3.0	21.4±2.8
ASA grade	13	14	13
IASA grade II	12	11	12
Operation time (min)	89.9±3.5	90.5±5.0	91.4±4.7
Duration of anesthesia (min)	32.2±3.1	33.5±3.8	34.2±4.6

### Therapeutic methods

Before surgery, all patients were intravenously injected with propofol (1.2 mg/kg) for anesthesia induction and then with sufentanil (0.5 μg/kg) and rocuronium (0.8 mg/kg). Next, double-lumen endotracheal intubation was conducted for smooth insertion, during which a fiberoptic bronchoscope was adopted to locate the tracheal tube and ensure good alignment. Thereafter, an anesthesia respirator was connected for mechanical ventilation (VT: 8 mL/kg, RR: 12 times/min, suction ratio: 1:2, inhaled oxygen concentration: 100%, oxygen flow rate: 1 L/min, P_ET_ CO_2_ : 40 mmHg), and the respiratory tract was kept clear for sufficient oxygen inhalation. Anesthesia was maintained by jointly injecting with propofol (5 mg/kg/h) and remifentanil (0.2 μg/kg/min), with rocuronium (0.3 mg/kg) added at intervals. Before surgery, the patients in group D1 were given dexmedetomidine at load capacity of 1 μg/kg using an infusion pump for 10 min and then at 0.5 μg/kg/h until the chest was closed. Those in group D2 were treated with dexmedetomidine at load capacity of 0.5 μg/kg using the infusion pump for 10 min and then at 0.3 μg/kg/h until the chest was closed. Those in group C were given the same amount of normal saline. The position of the patients was changed, and then the fiberoptic bronchoscope was aligned for OLV. The changes in various indexes were observed before induction (T0) and at 15 min after two-lung ventilation(T1) and 5 min (T2) and 30 min (T3) after OLV.

### Determination of changes in hemodynamic indexes [mean arterial pressure (MAP), heart rate (HR) and pulse oxygen saturation (SpO_2_)], S_ct_O_2_, PaO_2_ and intapulmonary shunt Qs/Qt

The changes in the HR, MAP and SpO_2 _of patients were recorded in each group before thoracotomy (T1) and at 30 min (T2) after OLV. Arterial blood was sampled for blood gas analysis, the PaO_2 _was recorded, the intrapulmonary shunt Qs/Qt was calculated, and the S_ct_O_2_ was recorded at corresponding time points. Qs/Qt% = (CcO_2_ - CaO_2_)/(CcO_2_ - CvO_2_). CcO_2_ = Hb × 1.39 × SaO_2_ + (PaO_2_ × 0.0031), PaO_2_ = FiO_2_ × (Pb - PH2O) - (PaCO2/0.8), CaO_2_ = (1.34 × Hb × SaO_2_) + (0.0031 × PaO_2_), CvO_2_ = (1.34 × Hb × SvO_2_) + (0.0031 × PaO_2_).

### Detection of serum inflammatory factors via enzyme-linked immunosorbent assay (ELISA)

After collecting venous blood (5 mL) into Eppendorf (Ep) tubes containing anticoagulant from arms, centrifugation was conducted at room temperature and 3000 g for 15 min, followed by collection of the supernatant. Next, the levels of serum inflammatory factors (IL-6, IL-8 and TNF-α) were measured according to the instruments of the ELISA kit (Nanjing Jiancheng Bioengineering Institute, Nanjing, China). Later, the absorbance in each group was read using a microplate reader.

### Measurement of serum oxidative stress indexes through ELISA

Venous blood (5 mL) was collected into Ep tubes containing anticoagulant from arms and centrifuged at room temperature and 3500 g for 15 min. Thereafter, the supernatant was collected, and the changes in the content of serum oxidative stress indexes [MDA, SOD and reactive oxygen species (ROS)] were determined according to the instruments of the ELISA kit (Nanjing SenBeiJia Biological Technology Co., Ltd., Nanjing, China). Lastly, the microplate reader was utilized to read the absorbance in each group.

### Examination of lung function

Side stream spirometry was adopted to monitor lung function indicators [lung dynamic compliance (Cdyn), platform pressure (Pplat), and airway peak pressure (Ppeak)]. The average was taken after multiple measurements. The specific operations were performed as per the instructions of the instrument, and the obtained values were analyzed according to the instructions provided by manufacturers.

### Statistical analysis

All raw experimental data recorded were processed by Statistical Product and Service Solutions (SPSS) 19.0 analysis software (SPSS Inc., Chicago, IL, USA) and subjected to multiple comparisons. The experimental results obtained were expressed as mean ± standard deviation (x̅±SD), and P<0.05 suggested that the difference was statistically significant. Graphpad Prism 5.0 (La Jolla, CA, USA) was applied for plotting histograms.

## Results

### Hemodynamic indexes detected

As shown in Table 2, the MAP, HR and SpO_2_ exhibited no obvious differences at each observation time point among three groups (P>0.05), suggesting that there is no impact on hemodynamics during OLV.

**Table 2:** Hemodynamic indexes Note: No evident differences are detected in the MAP, HR and SpO_2_ among the three groups at each observation time point (P>0.05). MAP: mean arterial pressure, HR: heart rate; SpO_2_: pulse oxygen saturation.

Group	MAP	HR	SpO_2_ (%)
Group C at T0	79.9±1.0	81.5±1.1	89.1±0.8
T1	79.5±1.1	80.1±1.2	87.2±0.1
T2	79.0±1.2	81.4±1.1	85.8±0.4
T3	79.2±1.3	81.6±1.5	85.3±0.4
Group D1 at T0	78.8±1.1	80.9±1.0	85.7±0.9
T1	78.9±1.8	81.8±1.6	86.1±0.8
T2	78.5±1.9	80.5±1.7	85.1±0.6
T3	77.1±1.2	78.4±1.8	95.9±0.9
Group D2 at T0	78.8±1.3	79.5±1.2	85.3±0.8
T1	78.2±1.7	79.8±1.7	85.9±0.9
T2	80.2±3.0	79.7±2.1	84.5±0.3
T3	76.4±1.5	77.0±1.1	90.5±0.1

### Levels of inflammatory factors detected

At T2 and T3, the levels of serum IL-6, TNF-α and IL-8 showed marked decreases in group D1 and D2 compared with those in group C (P<0.05), and the decreases were overtly larger in group D1 than those in group D2, and they were evidently greater at T3 than those at T2 (P<0.05) (Table 3).

**Table 3:** Levels of serum IL-6, TNF-α and IL-8 Note: The levels of serum IL-6, TNF-α and IL-8 display remarkable decreases in group D1 and D2 compared with those in group C (P<0.05) at T2 and T3, and the decreases are overtly larger in group D1 than those in group D2, and they are evidently greater at T3 than those at T2 (P<0.05). Intra-group comparison: ^a^P<0.05 vs. T0, ^b^P<0.05 vs. T1, and ^c^p<0.05 vs. T2. Inter-group comparison: ^A^P<0.05 vs. group C, and ^B^P<0.05 vs. group D1.

Group	IL-8 (mg/L)	TNF-α	IL-6 (mg/L)
Group C at T0	58.9±1.7	38.6±1.0	46.8±1.9
T1	62.2±1.1	40.6±1.1	48.6±1.7
T2	65.7±1.9	42.5±1.3	49.4±1.6
T3	69.4±1.3	45.8±1.7	50.4±1.5
Group D1 at T0	59.9±1.5	40.3±1.1	46.9±1.7
T1	48.5±1.4^A^	38.1±1.4^A^	33.1±2.0^A^
T2	27.2±1.5^abA^	25.1±1.2^abA^	23.3±2.2^abA^
T3	21.6±1.1^abcA^	9.5±1.7^abcA^	10.5±2.5^abcA^
Group D2 at T0	58.7±1.9	41.2±1.7	47.8±1.4
T1	52.4±1.5^A^	37.2±1.6^A^	39.7±2.5^A^
T2	37.8±1.6^abAB^	29.4±1.4^abAB^	30.8±2.6^abAB^
T3	27.9±1.7^abcAB^	7.4±1.8^abcAB^	16.0±2.7^abcAB^

### Results of oxidative stress determination

According to Table 4, group D1 and D2 showed notably declined levels of serum ROS and MDA (P<0.05) and overtly raised SOD content (P<0.05) at T2 and T3 in comparison with group C, and the effects were markedly better in group D1 than those in group D2, and they were significantly superior at T3 to those at T2 (P<0.05).

**Table 4:** Content of serum ROS, MDA and SOD Note: Compared with those in group C, the levels of serum ROS and MDA are prominently decreased (P<0.05) in group D1 and D2 at T2 and T3, while the SOD content is remarkably elevated (P<0.05), and the effects are markedly better in group D1 than those in group D2, and they are significantly superior at T3 to those at T2 (P<0.05).Intra-group comparison: ^a^P<0.05 vs. T0, ^b^p<0.05 vs. T1, and ^c^P<0.05 vs. T2. Inter-group comparison: ^A^P<0.05 vs. group C, and ^B^P<0.05 vs. group D1.

Group	ROS (U/L)	MDA (mmol/L)	SOD (U/mg)
Group C at T0	30.5±1.4	16.5±1.8	4.1±1.0
T1	32.5±1.7	17.6±1.4	3.4±1.4
T2	33.7±1.8	18.2±1.1	3.8±1.5
T3	34.8±1.9	17.0±1.3	4.3±1.2
Group D1 at T0	31.1±1.8	17.2±1.4	4.2±1.8
T1	28.1±1.7^A^	15.2±1.0^A^	6.8±1.4^A^
T2	18.4±1.4^abA^	10.5±1.0^abA^	12.6±1.6^abA^
T3	7.6±1.9^abcA^	4.6±1.1^abcA^	21.5±1.5^abcA^
Group D2 at T0	31.5±1.3	17.9±1.8	4.0±1.6
T1	27.8±1.8^A^	16.2±1.4^A^	5.2±1.4^A^
T2	22.1±1.1^abAB^	13.5±1.3^abAB^	8.6±1.8^abAB^
T3	14.6±1.5^abcAB^	8.4±1.0^abcAB^	15.8±1.3^abcAB^

### S_ct_O_2_

Compared with that at T0, the S_ct_O_2_ in group D1 and D2 was evidently lowered at T2 and T3, and group D1 exhibited a smaller decrease than group D2 (P<0.05) (Table 5).

**Table 5:** S_ct_O_2_ in different groups Note: The S_ct_O_2_ is significantly lowered in group D1 and D2 at T2 and T3 compared with that at T0 and T1, and the decrease in group D1 is distinctly smaller than that in group D2 (P<0.05). Intra-group comparison: ^a^P<0.05 vs. T0, ^b^P<0.05 vs. T1, and ^c^P<0.05 vs. T2. Inter-group comparison: ^A^P<0.05 vs. group C, and ^B^P<0.05 vs. group D1.

Group	T0	T1	T2	T3
S_ct_O2 (%) Group C	81.5±1.5	70.5±1.0^a^	65.5±1.2^ab^	60.1±1.7^abc^
Group D1	82.1±1.1	80.7±1.6^A^	76.8±1.6^abA^	70.1±1.9^abcA^
Group D2	80.1±1.6	75.1±1.9^AB^	70.8±1.7^abAB^	65.1±1.3^abcAB^

### PaO_2_ and the intrapulmonary shunt Qs/Qt

As shown in Table 6, group D1 and D2 had notably lowered Qs/Qt (P<0.05) and overtly elevated PaO_2_ content (P<0.05) at T2 and T3 in comparison with group C, and the decline and increase were markedly greater in group D1 than those in group D2, and they were significantly larger at T3 than those at T2 (P<0.05).

**Table 6:** PaO_2_ and the intrapulmonary shunt Qs/Qt Note: The Qs/Qt is significantly lower in group D1 and D2 than that in group C at T2 and T3 (P<0.05), while the PaO_2_ content is notably raised (P<0.05), and such decrease and increase are significantly larger in group D1 than those in D2 group, and they are obviously greater at T3 than those at T2 (P<0.05). Intra-group comparison: ^a^P<0.05 vs. T0, ^b^P<0.05 vs. T1, and ^c^P<0.05 vs. T2. Inter-group comparison: ^A^P<0.05 vs. group C, and ^B^P<0.05 vs. group D1.

Group	PaO_2_ (mmHg)	Qs/Qt(%)
Group C at T0	80.5±1.5	31.5±1.7
T1	285.1±1.7^a^	32.4±1.5
T2	186.1±1.6^ab^	33.9±1.6
T3	152.6±1.9^abc^	31.0±1.1
Group D1 at T0	81.5±1.0	32.1±1.5
T1	185.4±1.9^aA^	30.1±1.2^A^
T2	100.8±1.4^abA^	15.4±1.1^abA^
T3	130. 9±1.5^abcA^	5.9±1.3^abcA^
Group D2 at T0	83.1±1.7	31.9±1.7
T1	180.4±1.5^aA^	29.1±1.5
T2	91.5±1.7^abAB^	20.1±1.8^abAB^
T3	110.6±1.9^abcAB^	10.4±1.9^abcAB^

### Lung function indexes detected

The results of lung function index detection (Table 7) revealed that the Cdyn, Pplat and Ppeak displayed no significant differences among three groups at T0 and T1. At T2 and T3, the Cdyn was evidently raised, while the Pplat and Ppeak overtly declined (P<0.05). Moreover, group D1 had better effects in comparison with group D2, and the effects were obviously superior at T3 to those at T2 (P<0.05).

**Table 7:** Lung function indexes detected Note: At T0 and T1, there are no significant differences in the Cdyn, Pplat and Ppeak among three groups. At T2 and T3, the Cdyn is significantly elevated, while the Pplat and Ppeak overtly decline (P<0.05), and group D1 has better effects in comparison with group D2, and the effects are obviously superior at T3 to those at T2 (P<0.05). Intra-group comparison: ^a^P<0.05 vs. T0, ^b^P<0.05 vs. T1, and ^c^P<0.05 vs. T2. Inter-group comparison: ^A^P<0.05 vs. group C, and ^B^P<0.05 vs. group D1.

Group	Cdyn(mL/cm H_2_O)	Pplat(cm H_2_O)	Ppeak(cm H_2_O)
Group C at T0	25.3±2.2	34.5±2.8	30.5±2.2
T1	26.3±2.7	33.4±2.4	29.4±2.8
T2	27.8±2.5	30.1±2.9	28.4±2.4
T3	28.1±2.9	29.8±2.0	27.6±2.5
Group D1 at T0	26.3±2.0	35.7±2.0	31.4±1.2
T1	28.4±2.8	30.4±2.7	30.1±1.8
T2	40.8±2.9^abA^	18.4±2.9^abA^	19.4±1.3^abA^
T3	52.7±2.3^abcA^	8.4±2.3^abcA^	9.1±1.3^abcA^
Group D2 at T0	25.9±2.1	35.1±2.4	31.8±2.0
T1	26.9±2.7	30.4±2.6	30.5±1.0
T2	34.8±2.1^abAB^	24.6±2.8^abAB^	25.8±1.6^abAB^
T3	44.8±2.4^abcAB^	16.7±2.0^abcAB^	17.6±1.9^abcAB^

## Discussion

Hypoxemia is caused bright-to-left shunt and uneven distribution of alveolar ventilation and pulmonary perfusion in lungs, and the high ventilation/perfusion area interferes in the effective clearance of CO_2_, which may result in hypercapnia [15]. OLV will aggravate intrapulmonary shunt and dead space, while gravity and HPV confer protective effects [16]. Besides, treatment strategies for OLV-induced hypoxemia, such as positive end-expiratory pressure ventilation and recruitment of alveoli, are not very effective in inhibiting the progression of the disease. In addition to the β-adrenergic receptors in bronchial smooth muscle, there are α1 and α2-adrenergic receptors expressed in the bronchial mucosa and ganglia [17]. The bronchodilators currently used target the β-adrenergic receptors in the bronchial wall, and the effects of the bronchodilators targeting the α-adrenergic receptors have not been verified. Dexmedetomidine, a selective α-adrenergic receptor agonist, is reported to effectively repress histamineinduced bronchoconstriction and reduce the intrapulmonary shunt in healthy patients during OLV in an animal study [18]. What's more, it is known that dexmedetomidine is capable of directly lowering pulmonary artery pressure, and will not increase pulmonary artery pressure in patients with pulmonary hypertension [19]. Vickovic et al. [20] found that magnesium sulfate as an adjuvant to anesthesia in patients with arterial hypertension reduces hemodynamic changes during anesthesia. It was found in this study that there were no evident differences in the MAP, HR and SpO_2_ among three groups at each observation time point, indicating that there is no influence on hemodynamics during OLV. ROS plays an important role in various tissue damage like liver damage. Reactive oxygen radicals have been associated with many diseases including autoimmune diseases like rheumatoid arthritis, diabetes mellitus, atherosclerosis, obesity, hypertension and cardiovascular diseases such as ischemia [21] [22]. It can also trigger a cascade of cell damage and necrosis/apoptosis and subsequent pro-inflammatory response, further facilitating the progression of diseases [23]. In this study, it was discovered that the serum IL-6, TNF-α and IL-8 levels were markedly down-regulated in group D1 and D2 compared with those in group C at T2 and T3, and the decreases were overtly larger in group D1 than those in group D2, and they were evidently greater at T3 than those at T2. Besides, the serum ROS and MDA levels were clearly reduced, while the SOD content obviously rose in group D1 and D2 at T2 and T3 compared with those in group C. Additionally, the effects were markedly better in group D1 than those in group D2, and they were significantly superior at T3 to those at T2.

In abdominal surgeries, anesthesia management based on brain saturation monitoring is able to shorten hospital stays and reduce cognitive dysfunction. In abdominal surgeries for the elderly, decreased cerebral blood oxygen level is almost always correlated with massive or continued hemorrhage and significantly down-regulated hemoglobin level [24]. The results of this study manifested that the S_ct_O_2_ was evidently lowered in group D1 and D2 at T2 and T3 compared with that at T0, and the decrease in group D1 was distinctly smaller than that in group D2. PaO_2_ triggers the contraction of capillaries by inhibiting the nitric oxide and cyclooxygenase pathways. Anesthetics and techniques may affect shunt by altering cardiac output, pulmonary vascular tone and modification of HPV. Furthermore, hemodynamic parameters and anesthesia needs are measured and evaluated as secondary outcomes, which may have effects on shunt [25] [26]. Elhakim et al [27]. studied the effect of infusion of dexmedetomidine and found that dexmedetomidine reduces the shunt and improves oxygenation, and patients receiving epidural anesthesia with dexmedetomidine have lowered bispectral index values, intraoperative awareness and need for analgesia. It was found in this study that at T2 and T3, the Qs/Qt was overtly lowered, while the PaO_2_ content was significantly elevated in group D1 and D2 compared with those in group C, and the effects were markedly better in group D1 than those in group D2, and they were significantly superior at T3 to those at T2. Moreover, an animal study revealed that dexmedetomidine increases pulmonary artery pressure and pulmonary vascular resistance via direct effects of its receptors on the vascular smooth muscle. Similar changes are also observed in healthy volunteers when the plasma concentration of dexmedetomidine infused reaches 1.9 ng/mL [27]. In this study, it was revealed that no significant differences were detected in the Cdyn, Pplat and Ppeak among three groups at T0 and T1. At T2 and T3, the Cdyn was notably raised, while the Pplat and Ppeak were overtly reduced, and group D1 had overtly better effects in comparison with group D2, and the effects were obviously superior at T3 to those at T2. The results of this study are similar to the findings of above studies.

## Conclusions

According to our results, the present study demonstrated that dexmedetomidine is able to effectively mitigate inflammatory response and oxidative stress, lower oxygenation and Qs/Qt and improve S_ct_O_2_ and lung function during OLV with good effects. Our study found that patients undergoing open pulmonary lobectomy and observing hemodynamic parameters, inflammatory factors, oxidative stress indicators, and changes in S_ct_O_2_, PaO_2_ and Qs/Qt as well as lung function at different time points, provides theoretical and experimental bases for the popularization and application of dexmedetomidine. Although our study provided a good experimental basis for the research and development of adrenergic receptor drugs, further studies in dexmedetomidine patients are still required for indications of antioxidative therapy during anaesthesia.

## Dodatak

### Acknowledgements

No.

### Conflict of interest statement

The authors reported no conflict of interest regarding the publication of this article.
